# BRD4/8/9 are prognostic biomarkers and associated with immune infiltrates in hepatocellular carcinoma

**DOI:** 10.18632/aging.103768

**Published:** 2020-09-14

**Authors:** Yi-Ru Chen, Su-Shan Ouyang, Yan-Ling Chen, Ping Li, Hui-Wen Xu, Sen-Lin Zhu

**Affiliations:** 1 Department of Gastroenterology and Hepatology, The First Affiliated Hospital of Sun Yat-sen University, Guangzhou, Guangdong Province, China; 2 Department of Anesthesiology, Hospital of Stomatology, Guanghua School of Stomatology, Sun Yat-sen University, Guangzhou, China, Guangdong Provincial Key Laboratory of Stomatology, Sun Yat-sen University, Guangzhou, Guangdong Province, China

**Keywords:** bromodomain, BRD, hepatocellular carcinoma, prognosis, immune infiltrates

## Abstract

Bromodomain (BRD)-containing proteins are a class of epigenetic readers with unique recognition for N-acetyl-lysine in histones and functions of gene transcription and chromatin modification, known to be critical in various cancers. However, little is known about the roles of distinct BRD-containing protein genes in hepatocellular carcinoma (HCC). Most recently, we investigated the transcriptional and survival data of BRD1, BRD2, BRD3, BRD4, BRD7, BRD8, BRD9 in HCC patients through ONCOMINE, UALCAN, Human Protein Atlas, GEPIA, cBioPortal, STRING, TIMER databases. BRD1/2/3/4/7/8/9 were over-expressed in HCC and were significantly associated with clinical cancer stages and pathological tumor grades. High mRNA expressions of BRD4/8/9 were promising candidate biomarkers in HCC patients. The rate of sequence alternations in BRD1/2/3/4/7/8/9 was relatively high (52%) in HCC patients, and the genetic alternations were correlated with shorter overall survival and disease-free survival in HCC patients. Additionally, the mRNA expression levels of individual BRD genes were significantly positively associated with the immune infiltrating levels of B cells, CD8^+^ T cells, CD4^+^ T cells, macrophages, neutrophils, and dendritic cells. And the associations between BRD1/2/3/4/7/8/9 and diverse immune marker sets showed a significance. Overall, these results indicated that BRD4/8/9 could be potential prognostic markers and druggable epigenetic targets in HCC patients.

## INTRODUCTION

Hepatocellular carcinoma (HCC) is the fifth most prevalent malignancy worldwide and ranked the second-highest cancer mortality rate [1, 2]. Despite great efforts in HCC diagnosis and treatment, the prognosis of HCC remains poor, and less than 9% of HCC patients could survive for more than five years [3]. Though researchers have considered epigenetic and genetic alternations as some of the dominant elements in HCC [4], the underlying pathogenesis and etiology on the molecular level of HCC are still obscure. Further studies of HCC oncogenes would help to identify new molecular markers of HCC progression and improve diagnostic and therapeutic options.

Acetylation of lysine residues on histone tails is one of the major epigenetic modifications relating to transcriptional activation. The bromodomain (BRD), an evolutionarily well-conserved domain of approximately 110 amino acids, is the first protein module known to be able to specifically recognize and bind to N-acetyl-lysine of the histones [5–8]. The human genome encodes 61 BRDs in 46 nuclear and cytoplasmic proteins. Based on sequence and function homology, this protein family could be divided into eight subgroups [9]. BRD-containing proteins play important roles in gene transcription, including serving as transcription factors, transcriptional co-regulators, or major recruiting factors for the assembly of larger protein complexes. Moreover, they could exert catalytic activities, such as ATP-dependent chromatin remodeling, histone acetyltransferase (HAT), methyltransferase [10] (Supplementary Table 1). Therefore, BRD-containing proteins are transcriptional regulators that can launch chromatin remodeling in preparation for transcription.

Complex interactions between tumor and host, enacted by innate and adaptive immune cells and cytokines, profoundly affect the progression and prognosis of cancers [11]. Tumor infiltrations of neutrophils [12], tumor-associated macrophages (TAMs) [13] and regulatory T cells (Tregs) [14] are associated with poor prognosis in HCC patients while infiltrating B cells [15], CD8^+^ cytotoxic T lymphocytes (CTLs) [16] and dendritic cells (DCs) [17] predict favorable clinical outcome of HCC. Moreover, elevated T-helper 17/T-helper 1 (Th17/Th1) ratio may promote HCC progression [18] while elevated T-helper 1/T-helper 2 (Th1/Th2) ratio is correlated with a good prognosis [19]. Recently, several studies have discovered that BRD-containing proteins may affect the immune microenvironment [20–22]. For example, BRD2 and BRD4 controlled Th17 cell differentiation in a bromodomain-dependent manner by directly binding to the *IL17* genomic locus [21].

Given the important roles of BRD-containing proteins in regulating gene transcription and chromatin remodeling, alternations (mutations, over-expression, deletions, translocations, etc.) of these proteins have reportedly a close relationship with the tumorigenesis and progression of various types of cancer. Recent studies have found aberrant expressions and their prognostic values in some BRD-containing protein genes in HCC. For example, BRD4 was significantly up-regulated in HCC tissues compared with adjacent normal tissues. And the forced expression of BRD4 was sufficient to promote tumor growth and epithelial-mesenchymal transition in HCC [23]. Significantly down-regulation of BRD7 had been found in HCC tissues and HCC cell lines, and higher BRD7 expression was correlated with a higher survival rate [24]. Nevertheless, the roles of distinct BRD-containing protein genes in the development and progression of HCC remained unknown. In the current study, we aimed to address this question through in-depth analysis of the expressions, mutations and predictive signaling pathways of 7 members from BRD gene family, namely BRD1, BRD2, BRD3, BRD4, BRD7, BRD8 and BRD9, and their associations with prognosis and immune infiltrates in HCC patients.

## RESULTS

### Over-expression of BRD-containing protein genes in HCC patients

**Figure 1:**
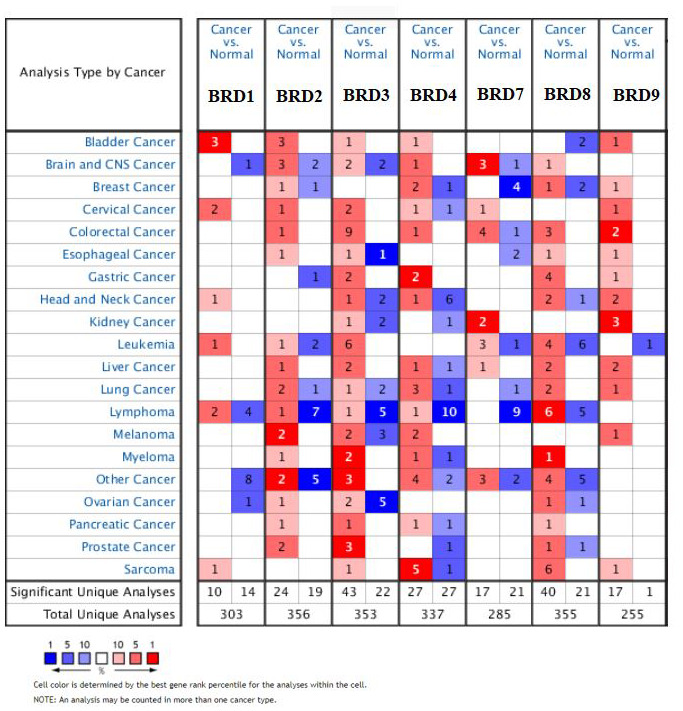
**Transcription levels of 7 BRD-containing protein genes in different types of cancers (ONCOMINE database).** The numbers in colored cells show the quantities of datasets with statistically significant mRNA overexpression (red) or downexpression (blue) of target genes. Cell color was determined by the best gene rank percentile for the analysis within the cells. The number in each cell represents the number of analyses that satisfied the threshold, such as gene rank percentile (10%), *P*-value (0.001), and fold change (1).

Seven BRD-containing protein genes have been identified in the human genome, and their transcriptional expression and proteinic expression have been determined using the ONCOMINE database, UALCAN and Human Protein Atlas. As shown in Figure 1 and Table 1, the mRNA expression levels of seven BRD genes in 20 types of cancers compared to normal tissues were firstly examined by ONCOMINE. Noticeably, except for BRD1, BRD2/3/4/7/8/9 showed a commonly up-regulated expression pattern in HCC samples (fold-change: > 1, *P*-value < 0.001). BRD2 and BRD7 mRNA expressions showed 1.726-fold (*P* = 7.90E-6) and 1.446-fold (*P* = 7.75E-5) elevation from Wurmbach Liver dataset in liver samples [25], respectively. In Chen Liver dataset [26], BRD3 up-regulation was found in HCC samples compared to normal tissues with a fold change of 1.523 (*P* = 4.52E-11) while Wurmbach observed 1.592-fold elevation [25] (*P* = 2.24E-4). Similarly, BRD4 expression showed 1.781-fold elevation in Roessler Liver dataset [27] (*P* = 4.69E-7). BRD8 mRNA expression showed significant elevation in HCC tissues in Wurmbach Liver dataset [25] with a fold change of 1.517 (*P* = 5.95E-7) and Chen Liver dataset [26] with a fold change of 1.406 (*P* = 1.99E-9), respectively. In comparison with normal tissues, BRD9 mRNA expression in HCC samples exhibited 1.526-fold elevation in Roessler Liver dataset [27] (*P* = 1.14E-7) and 1.462-fold increase in Wurmbach Liver dataset [25] (*P* = 9.67E-5).

**Table 1:** Significant changes of 7 BRD-containing protein genes expression in mRNA level between HCC and normal liver tissues (ONCOMINE).

	**Fold Change**	***P* value**	***t*-test**	**Reference**
BRD1				
	NA	NA	NA	NA
BRD2				
	1.726	7.90E-6	5.096	Wurmbach Liver
BRD3				
	1.523	4.52E-11	6.902	Chen Liver
	1.592	2.24E-4	4.327	Wurmbach Liver
BRD4				
	1.781	4.69E-7	5.791	Roessler Liver
BRD7				
	1.446	7.75E-5	4.356	Wurmbach Liver
BRD8				
	1.517	5.95E-7	5.730	Wurmbach Liver
	1.406	1.99E-9	6.205	Chen Liver
BRD9				
	1.526	1.14E-7	6.493	Roessler Liver
	1.462	9.67E-5	4.452	Wurmbach Liver

**Figure 2:**
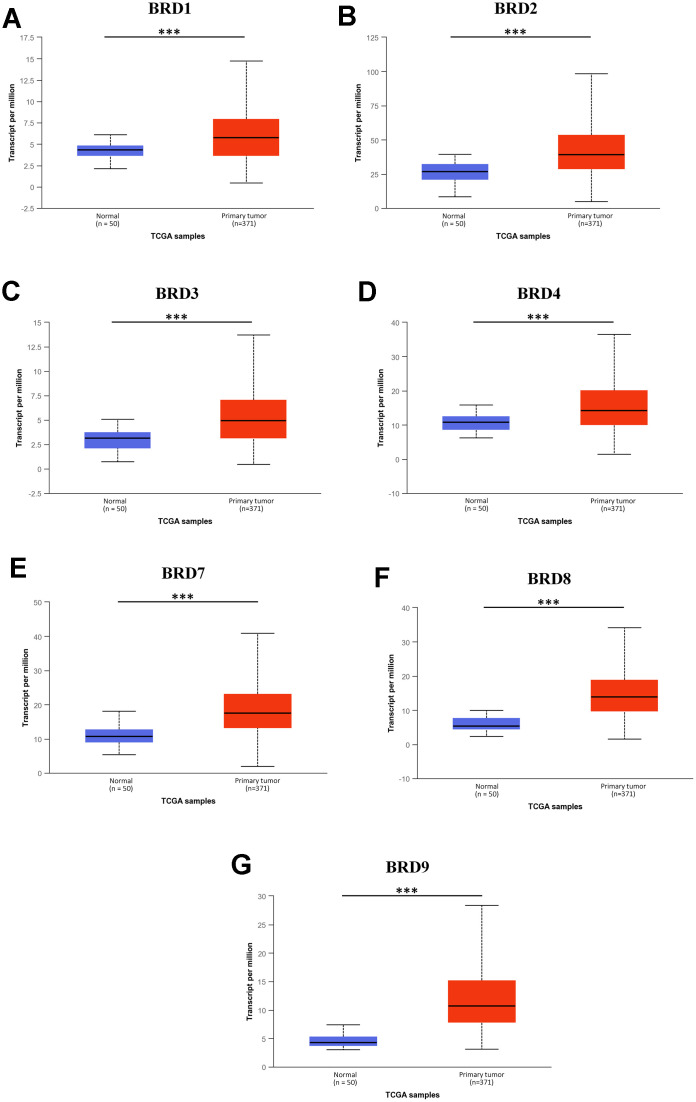
**mRNA expression of 7 distinct BRD-containing protein genes in HCC tissues and adjacent normal liver tissues from TCGA database (UALCAN).** mRNA expressions of 7 BRD-containing protein genes were over-expressed in primary HCC tissues compared to normal tissues (**A**–**G**). ***P < 0.001.

**Figure 3:**
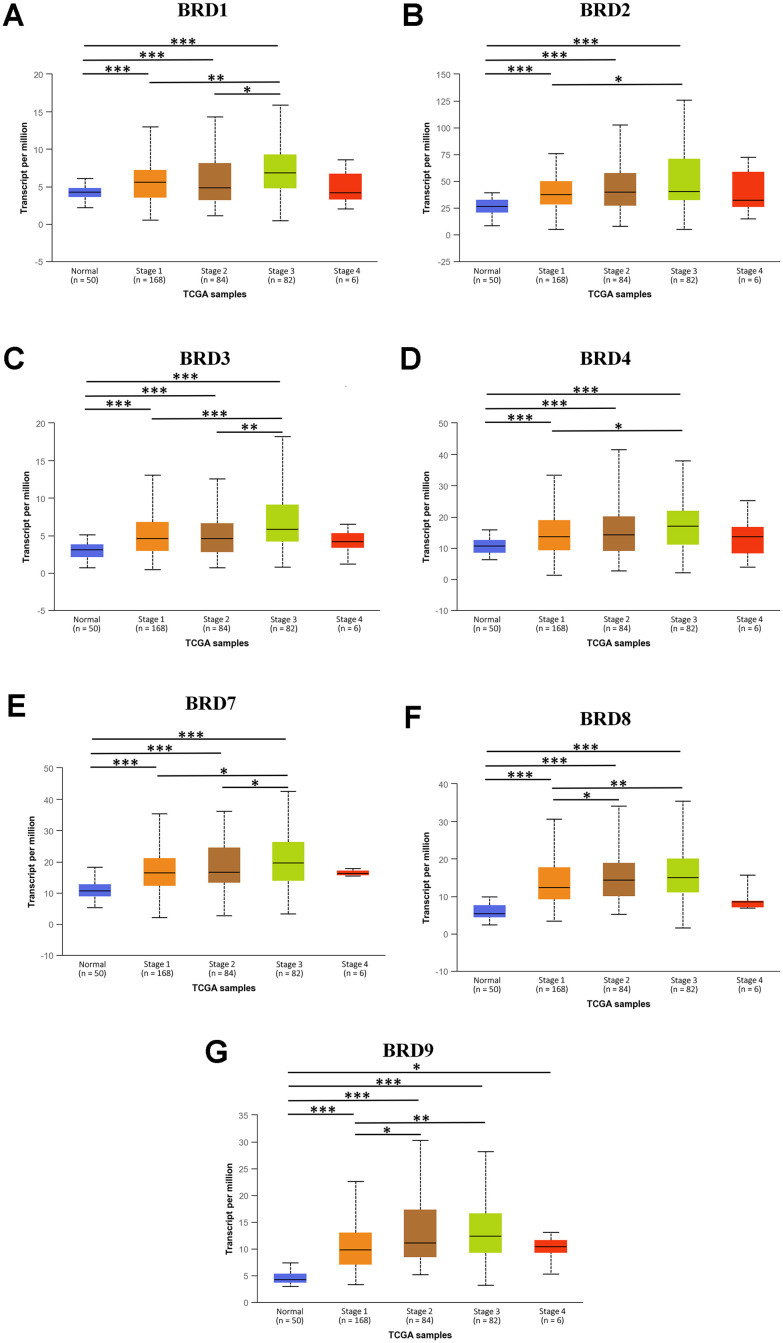
**The association between mRNA expression of 7 distinct BRD-containing protein genes and individual HCC cancer stages from TCGA database (UALCAN).** mRNA expressions of 7 BRD-containing protein genes were significantly associated with patients’ individual cancer stages. Patients with a more advanced stage of HCC had a tendency to express higher mRNA levels. The highest mRNA expressions of BRD1/2/3/4/7/8/9 were observed in stage 3 (**A**–**G**). *P < 0.05, **P < 0.01, ***P < 0.001.

**Figure 4:**
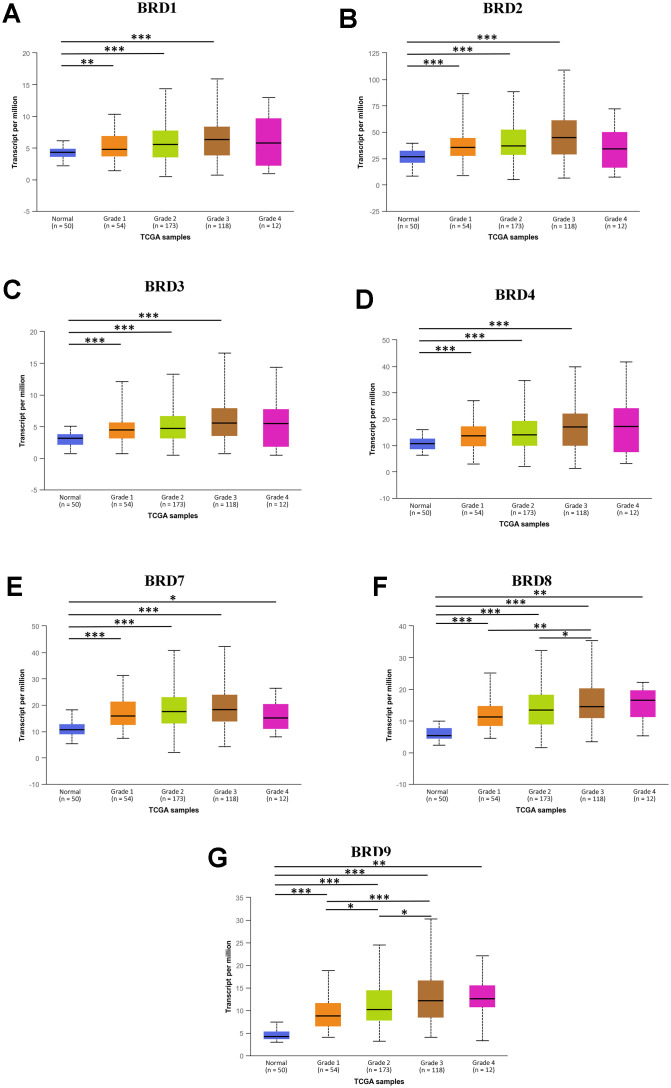
**The association between mRNA expression of 7 distinct BRD-containing protein genes and HCC tumor grades from TCGA database (UALCAN).** mRNA expressions of 7 BRD-containing protein genes were remarkably correlated with tumor grades. Along with the severity of histological grade in HCC, the mRNA expressions of 7 BRD-containing protein genes elevated. The highest mRNA expressions of BRD4/8/9 were found in tumor grade 4 (**D**, **F**, **G**), while the highest mRNA expressions of BRD1/2/3/7 were found in tumor grade 3 (**A**–**C**, **E**). *P < 0.05, **P < 0.01, ***P < 0.001.

Next, the mRNA expression levels of 7 BRD-containing protein genes between HCC and liver tissues were also detected using the UALCAN portal. The results showed higher mRNA expressions of BRD1/2/3/4/7/8/9 in primary HCC tissues than in normal tissues (Figure 2, all *P* < 0.05). The relationship of mRNA expressions of distinct BRD genes with clinicopathological parameters (including cancer stages and tumor grades in individual patients) was also analyzed. As shown in Figure 3, when classified by clinical stages, 7 BRD-containing protein genes were remarkably over-expressed in all stage subgroups compared to normal tissues, and patients with a more advanced stage of HCC tended to express higher mRNA levels. Patients in stage 3 expressed the highest mRNA expression levels. However, no statistical significance was found between the stage 4 group and the other groups, probably owing to a small sample size in stage 4. Consistently, as shown in Figure 4, the mRNA expressions of 7 BRD-containing protein genes elevated along with the severity of histological grade in HCC. Taken together, these results indicated that the mRNA levels of BRD1/2/3/4/7/8/9 were commonly up-regulated in HCC and correlated with cancer stages and histological grades in HCC patients.

**Figure 5:**
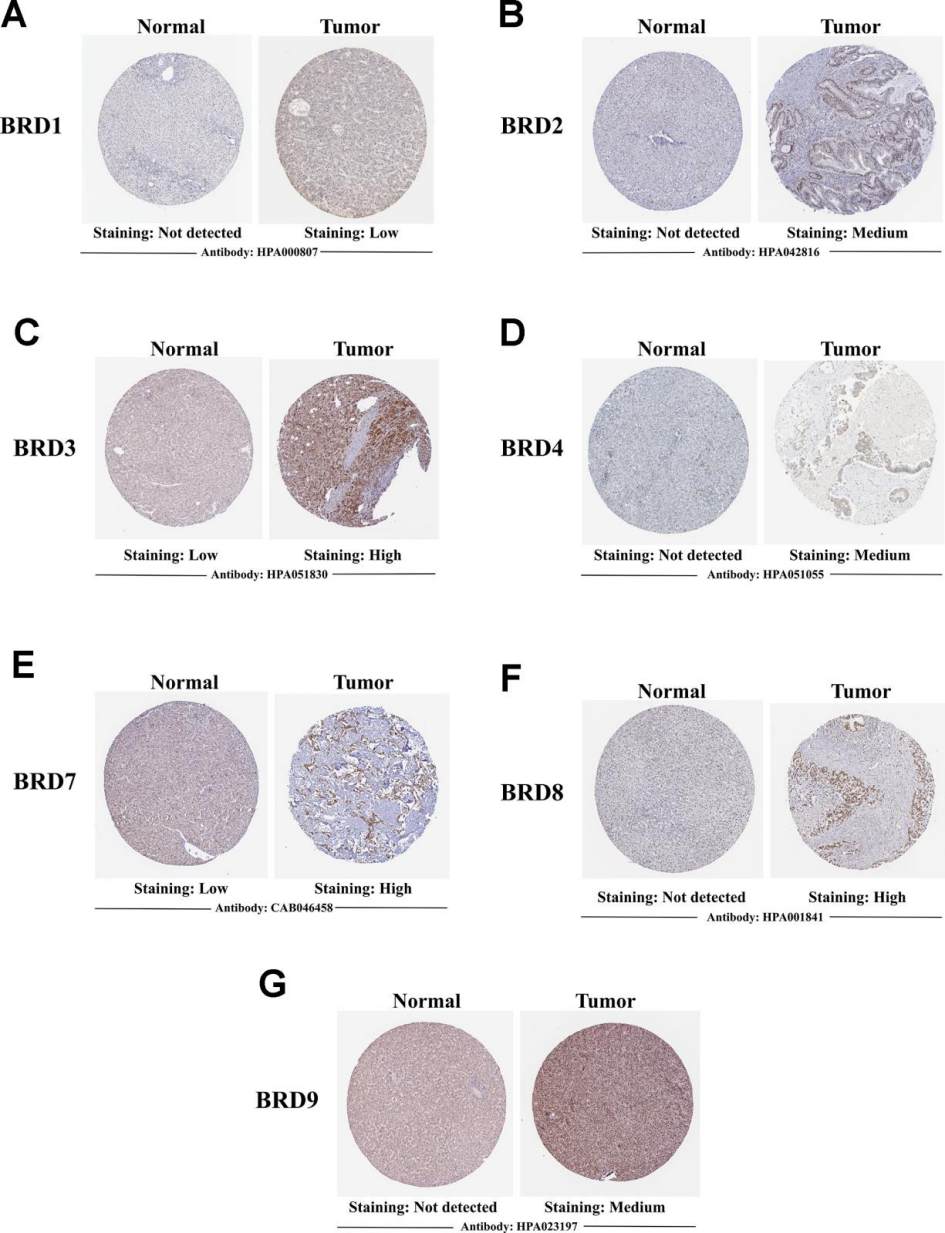
**Representative immunohistochemistry images of 7 distinct BRD-containing proteins in HCC tissues and normal liver tissues (Human Protein Atlas).** BRD1/2/4/8/9 were not expressed in normal liver tissues, while their low and medium protein expressions were observed in HCC tissues (**A**, **B**, **D**, **F**, **G**). Low expressions of BRD3/7 were observed in normal liver tissues, while their medium and high protein expressions were found in HCC tissues (**C**, **E**).

A comparative investigation of protein expression patterns of 7 BRD-containing proteins in HCC was performed using the Human Protein Atlas. As shown in Figure 5, BRD1/2/4/8/9 were not expressed in normal liver tissues, while their protein expressions were observed low- and medium-expressed in HCC tissues (Figure 5A, 5B, 5D, 5F, 5G). BRD3/7 were observed low-expressed in normal tissues, while their protein expressions were found medium- and high-expressed in HCC tissues (Figure 5C, 5E). In short, these results suggested that mRNA and protein expressions of BRD1/2/3/4/7/8/9 were commonly over-expressed in HCC patients.

### Prognostic values of BRD-containing protein genes in HCC patients

**Figure 6:**
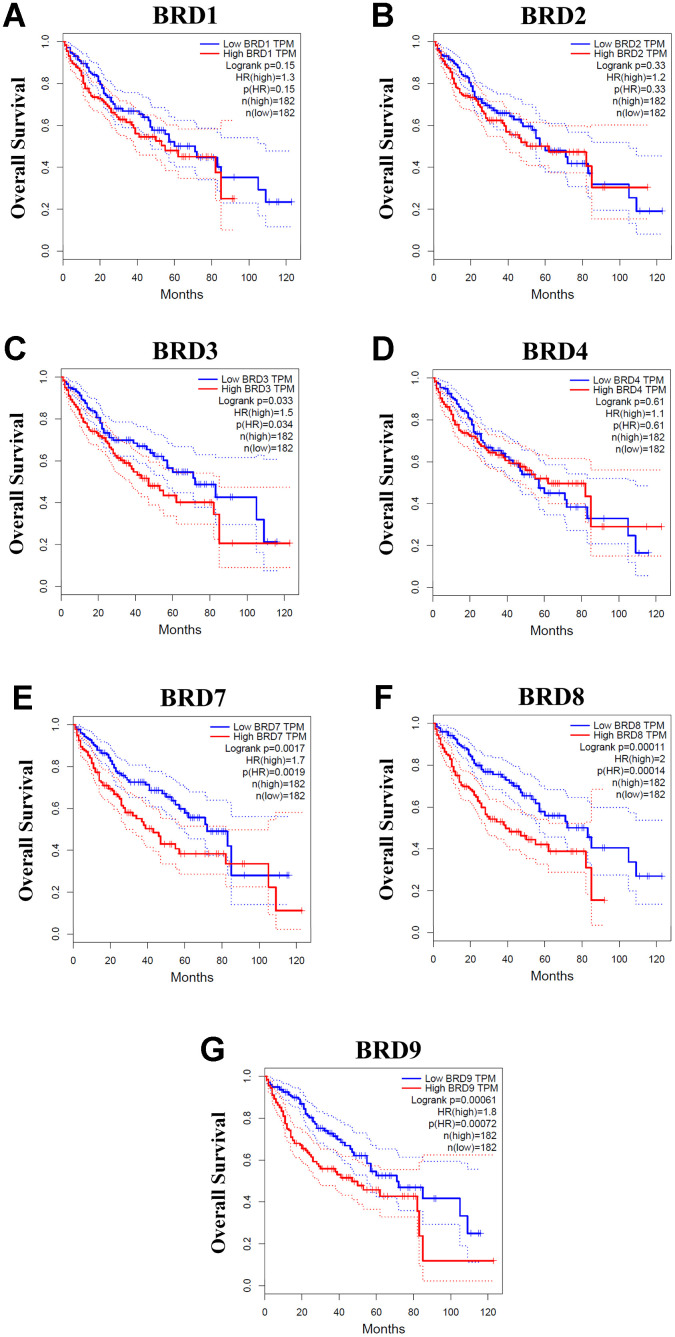
**Associations between mRNA expression of each BRD-containing protein gene in tumor tissues and OS of HCC patients (GEPIA).** Each mRNA expression of BRD-containing protein gene in tumor tissue was stratified into high or low expression using the median expression value as the cut-off point. The corresponding P-value for Log-rank test in all HCC patients was showed. Higher mRNA expressions of BRD3/7/8/9 were significantly associated with poorer OS in HCC patients (**C**, **E**–**G**). However, mRNA expressions of BRD1/2/4 showed no correlation with prognosis in HCC patients (**A**, **B**, **D**).

Using GEPIA, we next evaluated the prognostic values of the mRNA expressions of 7 BRD-containing protein genes in HCC patients. The patients were separated into high and low expression groups based on the median value. As shown in Figure 6, higher mRNA expressions of BRD3/7/8/9 were significantly associated with unfavorable overall survival (OS) of HCC patients (Figure 6C, 6E–6G). However, no significant correlation was observed between mRNA expressions of BRD1/2/4 and OS of HCC patients (Figure 6A, 6B, 6D). Moreover, the patients with higher BRD7/8/9 expression levels had worse disease-free survival (DFS) than those with lower BRD7/8/9 expression levels (Supplementary Figure 1E–1G). In contrast, BRD1/2/3/4 expressions showed no correlation with DFS in HCC patients (Supplementary Figure 1A–1D).

In the present study we also conducted further research to investigate the independent prognostic values of BRD-containing protein genes for OS and DFS of HCC patients. The clinicopathological data and mRNA sequencing of the BRD genes of 364 patients in TCGA liver cancer data were downloaded from the cBioPortal. Variables which showed a correlation with *P* ≤ 0.1 in univariate analysis were included into the multivariate Cox regression analysis. Univariate analysis for OS demonstrated that vascular invasion (HR = 1.384, 95% CI: 1.001-1.914, and *P* = 0.050), a more advanced pathologic stage (HR = 1.660, 95% CI: 1.355-2.035, and *P* < 0.001), high mRNA expressions of BRD7 (HR = 1.472, 95% CI: 1.040-2.083, and *P* = 0.029), BRD8 (HR = 1.913, 95% CI: 1.344-2.723, and *P* < 0.001) and BRD9 (HR = 1.686, 95% CI: 1.188-2.394, and *P* = 0.003), were correlated with unfavorable OS (Supplementary Table 3). Multivariate analysis further suggested that high BRD8 expression (HR = 1.545, 95% CI: 1.007-2.369, and *P* = 0.046) was significantly associated with unfavorable OS in HCC patients (Supplementary Table 4). Moreover, in univariate Cox regression analysis for DFS, HCC patients with vascular invasion (HR = 1.687, 95% CI: 1.288-2.210, and *P* < 0.001), high pathologic stage (HR = 1.731, 95% CI: 1.450-2.066, and *P* < 0.001), and high mRNA expressions of BRD2 (HR = 1.479, 95% CI: 1.092-2.003, and *P* = 0.011), BRD4 (HR = 1.608, 95% CI: 1.187-2.177, and *P* = 0.002), BRD8 (HR = 1.905, 95% CI: 1.404-2.585, and *P* < 0.001) and BRD9 (HR = 1.794, 95% CI: 1.323-2.434, and *P* < 0.001), had worse DFS (Supplementary Table 3). And multivariate analysis for DFS showed that high mRNA expressions of BRD4 (HR = 1.609, 95% CI: 1.119-2.313, and *P* = 0.010), BRD8 (HR = 1.703, 95% CI: 1.191-2.435, and *P* = 0.004) and BRD9 (HR = 1.590, 95% CI: 1.103-2.291, and *P* = 0.013) were independently correlated with worse DFS (Supplementary Table 5). Consequently, via multivariate survival analysis, BRD4/8/9 expressions were independently associated with worse DFS, and BRD8 was also independently correlated with unfavorable OS, suggesting BRD4/8/9 were promising prognostic factors in HCC patients although BRD4 and BRD9 were not independent predictors of OS in HCC patients.

### Genetic alternations in BRD-containing protein genes affect OS and DFS in HCC patients

**Figure 7:**
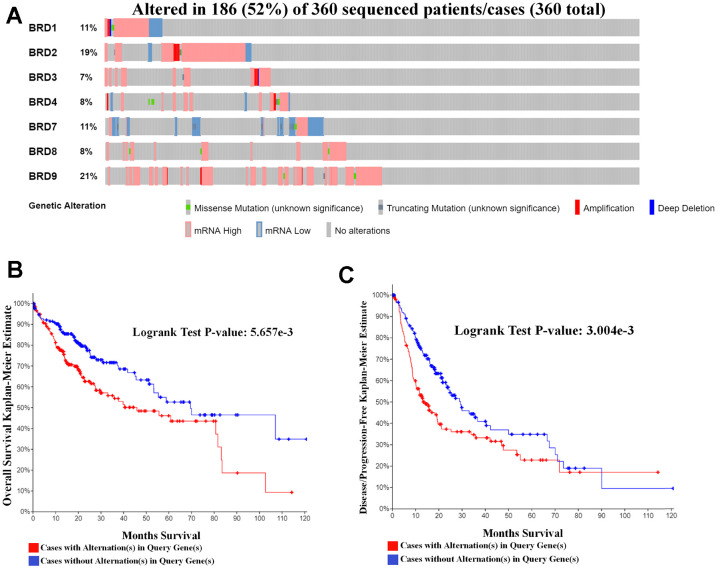
**Genetic mutations in BRD-containing protein genes and their relationship with OS and DFS in HCC patients (cBioPortal).** (**A**) Oncoprint in cBioPortal showed the distribution and proportion of samples with alternations in BRD-containing protein genes. A high rate (52%) of sequence alternations in BRD-containing protein genes was observed in 360 HCC patients. (**B**) Genetic alternations in BRD-containing protein genes were correlated with worse OS in HCC patients. (**C**) Genetic alternations in BRD-containing protein genes were correlated with worse DFS in HCC patients.

Further, the cBioPortal tool was applied to explore genetic alternations in BRD-containing protein genes and their correlations with OS and DFS in HCC patients. As shown in Figure 7A, BRD-containing protein genes were altered in 186 samples out of the 360 patients with HCC (the total rate of sequence alternations was 52%). BRD9, BRD2, BRD1 and BRD7 were the highest four genes with genetic alternations, and their rates were 21%, 19%, 11% and 11%, respectively. Moreover, a Kaplan-Meier plot and log-rank test demonstrated that the genetic alternations in BRD-containing protein genes were correlated with worse OS (Figure 7B, *P* = 5.657E-3) and DFS (Figure 7C, *P* = 3.004E-3). In short, these results indicated that the genetic alternations in BRD-containing protein genes could play a critical role in HCC patients’ prognosis.

### Functional protein interaction network of BRD-containing protein genes

Using the STRING database, a functional protein interaction network of BRD-containing protein genes was constructed to investigate the possible BRD-containing protein-mediated signaling pathways in HCC. As shown in Figure 8A, ten functional partners with the highest interacting confident scores were placed in the outer shell of the network, including KAT5, KAT6B, KAT7, MRGBP, YEATS4, MORF4L1, MORF4L2, PBRM1, ARID2 and SMARCE1. The other 40 interacting partners were placed in the inner shell with a confidence score above 0.9. Furthermore, DAVID was used to perform GO and KEGG analyses to find the functional enrichment of these 50 interactors based on the STRING database. As shown in Figure 8B, biological processes included chromatin remodeling; transcription, DNA-templated; histone H2A acetylation; covalent chromatin modification; and histone H4 acetylation. Cellular components analysis indicated that these protein localized mainly to the NuA4 histone acetyltransferase complex; nucleoplasm; SWI/SNF complex; npBAF complex; and nBAF complex (Figure 8C). Molecular function analysis found that these proteins were primarily involved in chromatin binding; transcription coactivator activity; RNA polymerase II distal enhancer sequence-specific DNA binding; nucleosomal DNA binding; and histone acetyltransferase activity (Figure 8D). As shown in Figure 8E, cell cycle, Notch signaling pathway, transcriptional misregulation in cancer, viral carcinogenesis, Wnt signaling pathway, pathways in cancer, and alcoholism were associated with tumorigenesis and development of HCC (Supplementary Table 6).

**Figure 8:**
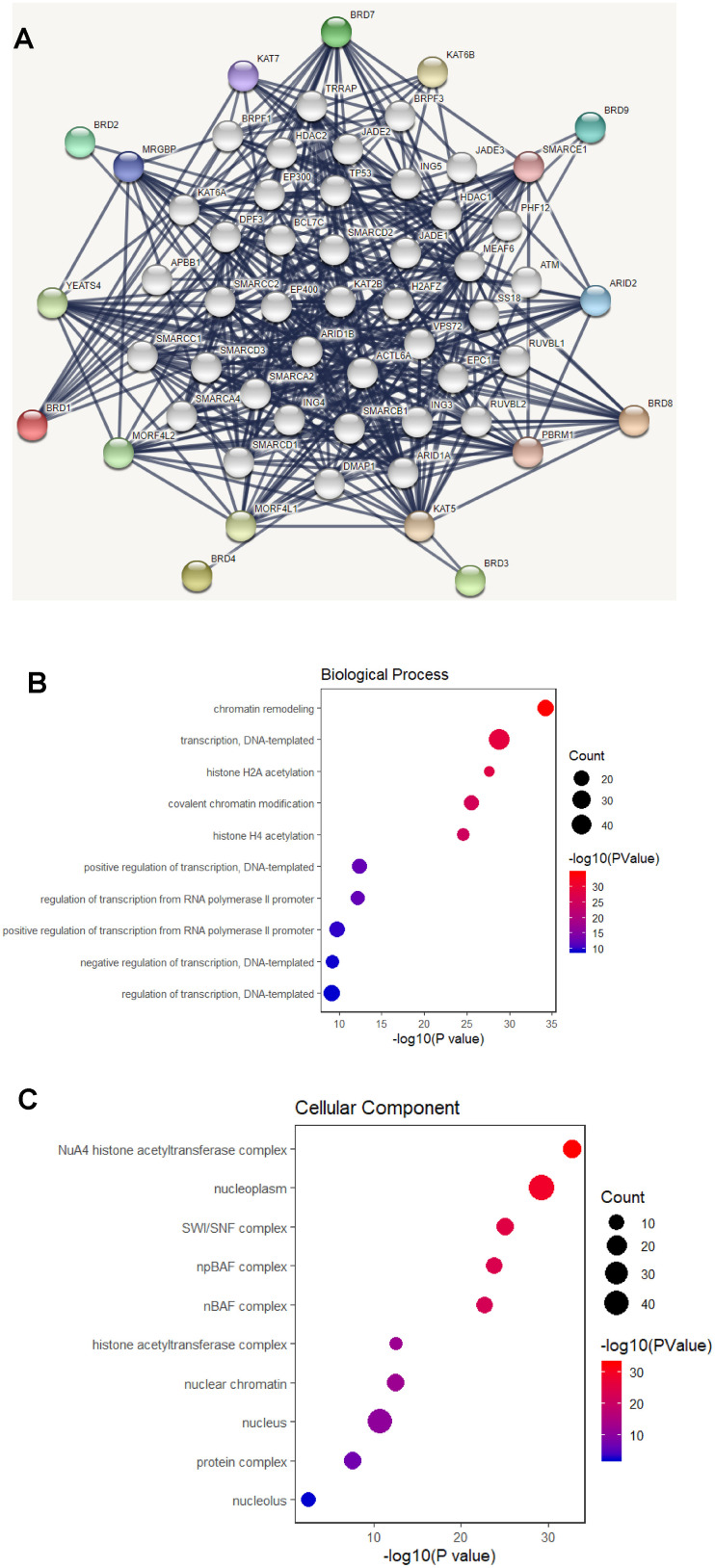
**Functional protein interaction network of BRD-containing protein genes (STRING and DAVID).** (**A**) Protein interaction network of 50 functional partners with confidence score > 0.9 based on STRING database. BRD 1/2/3/4/7/8/9 are the seed genes. Ten interacting partners with the highest confident scores were colored and placed in the outer shell. The other forty interacting partners were grey and placed in the inner shell. The blue lines represent the correlation between proteins and the thickness of the lines indicates the strength of data support. (**B**–**C**) The bubble diagram displayed the GO functional enrichment results of the 50 functional interactors, including biological process and cellular components.

**Figure 8:**
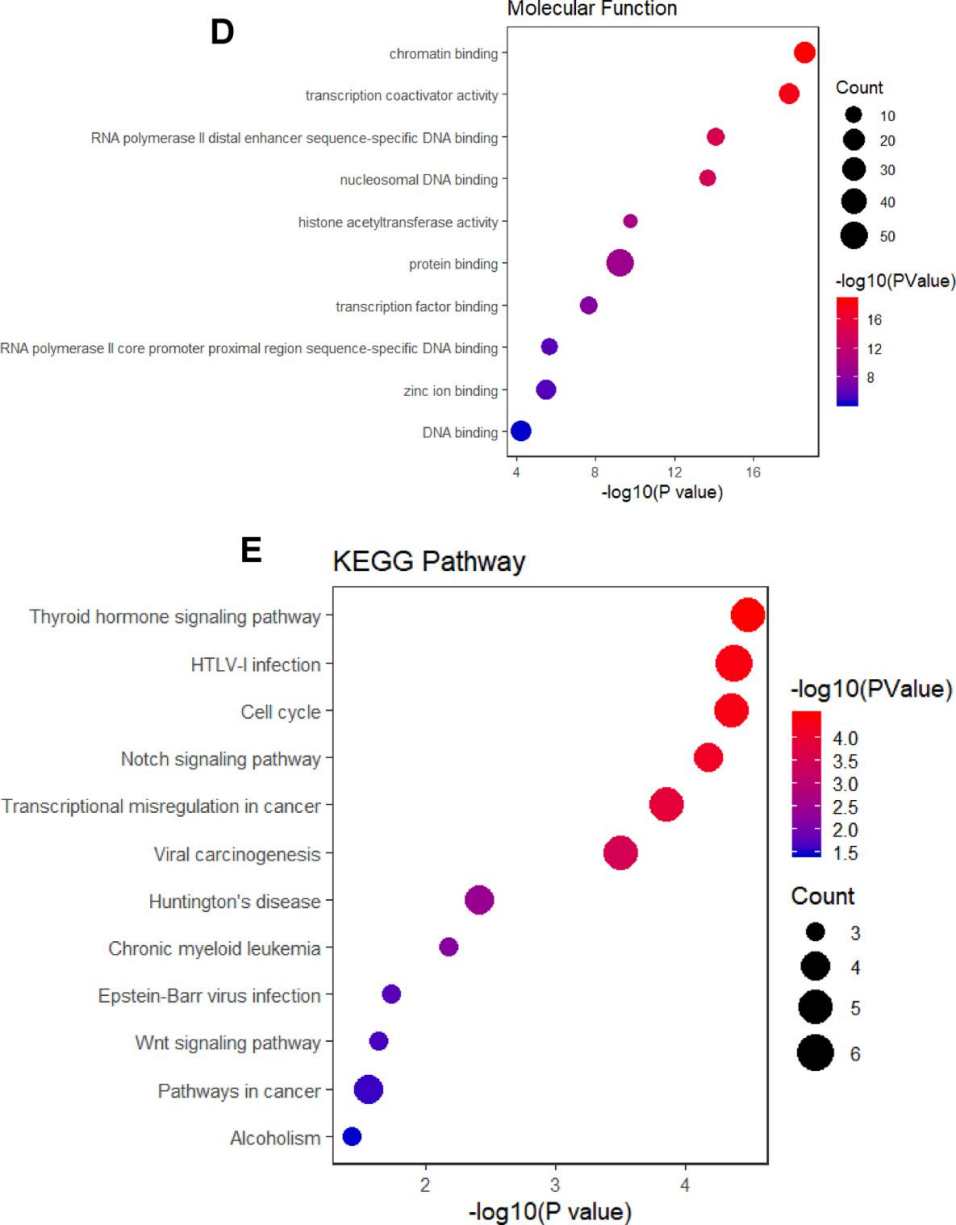
**Functional protein interaction network of BRD-containing protein genes (STRING and DAVID).** (**D**) The bubble diagram displayed the GO functional enrichment results of the 50 functional interactors, including molecular functions. (**E**) The bubble diagram showed the KEGG pathway analysis of the 50 functional interactors.

### Correlations between BRD-containing protein genes and immune infiltration levels

**Figure 9:**
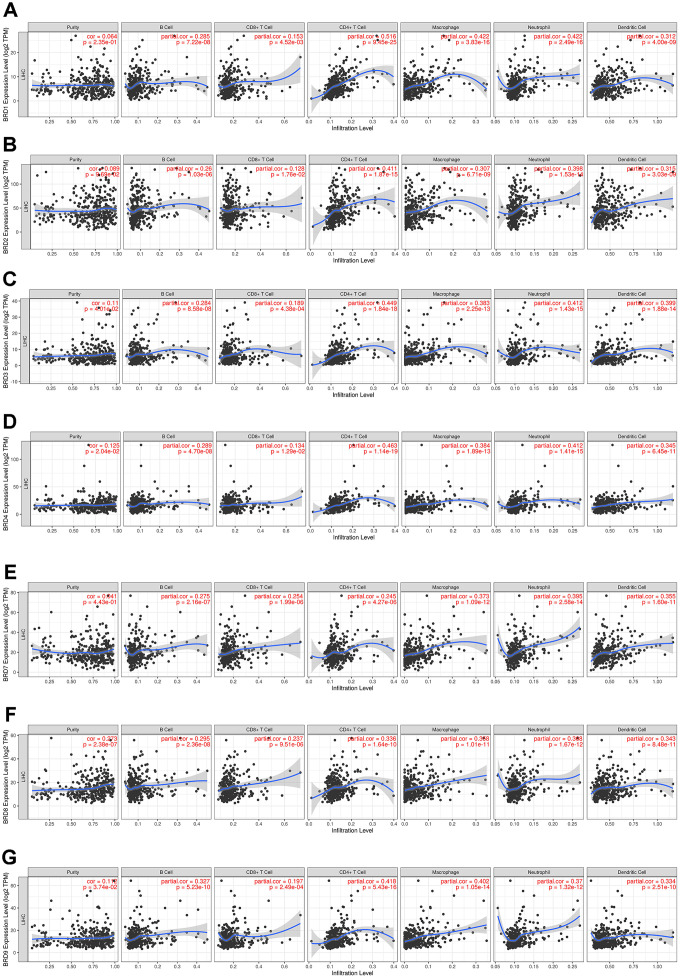
**Association of 7 BRD-containing protein genes with immune infiltrate levels in HCC (TIMER).** BRD1/2/3/4/7/8/9 expression had significant positive correlations with infiltrating levels of B cells, CD8+ T cells, CD4+ T cells, macrophages, neutrophils, and dendritic cells (n = 371) (**A**–**G**).

The TIMER website was applied to explore the associations between BRD-containing protein genes and the abundance of immune infiltrates in HCC patients. Noticeably, as shown in Figure 9, the mRNA expression levels of individual BRD-containing protein genes were significantly positively associated with the immune infiltrating levels of B cells, CD8^+^ T cells, CD4^+^ T cells, macrophages, neutrophils, and DCs. Further analysis of the correlations between BRD-containing protein genes and gene markers of different immune cells indicated that these 7 BRD genes were almost significantly associated with various gene markers of tumor-infiltrating immune cells, including PAX5, BCL6 and CD19 of B cells; CD8A, CTLA4 and LAT of CTLs; STAT4, TBX21 and CD4 of Th1 cells; GATA3, CXCR4 and CCR4 of Th2 cells; RORC, CCR6 and IL17A of Th17 cells; FOXP3, STAT5B and TGFB1 of Tregs; CCL2, IL10, CD163, VSIG4, CSF1R and FCGR2A of TAMs; ITGAX, CD1C and NRP1 of DCs; as well as CCR7, ITGAM and CD59 of neutrophils (Table 2). In short, these findings strongly indicated that BRD-containing protein genes played an important role in the HCC immune microenvironment.

**Table 2:** Correlation analysis between BRD-containing protein genes and gene markers of immune cells.

**Description**	**Gene marker**	**BRD1**		**BRD2**		**BRD3**		**BRD4**		**BRD7**		**BRD8**		**BRD9**	
		**Cor**	**P**	**Cor**	**P**	**Cor**	**P**	**Cor**	**P**	**Cor**	**P**	**Cor**	**P**	**Cor**	**P**
B cell	PAX5	0.195	***	0.195	***	0.254	***	0.198	***	0.147	**	0.12	*	0.071	0.189
	BCL6	0.466	***	0.506	***	0.484	***	0.449	***	0.403	***	0.375	***	0.269	***
	CD19	0.173	**	0.133	*	0.184	***	0.19	***	0.168	**	0.183	***	0.197	***
CTL	CD8A	0.212	***	0.244	***	0.263	***	0.231	***	0.178	***	0.216	***	0.188	***
	CTLA4	0.165	**	0.139	**	0.205	***	0.152	**	0.165	**	0.243	***	0.31	***
	LAT	0.19	***	0.148	**	0.167	**	0.16	**	0.126	*	0.247	***	0.27	***
Th1	STAT4	0.265	***	0.182	***	0.277	***	0.228	***	0.009	0.872	0.211	***	0.188	***
	TBX21	0.162	**	0.216	***	0.216	***	0.14	**	0.13	*	0.163	**	0.089	0.099
	CD4	0.281	***	0.213	***	0.308	***	0.302	***	0.259	***	0.26	***	0.151	**
Th2	GATA3	0.356	***	0.279	***	0.357	***	0.294	***	0.244	***	0.244	***	0.278	***
	CXCR4	0.494	***	0.416	***	0.506	***	0.419	***	0.358	***	0.363	***	0.37	***
	CCR4	0.533	***	0.482	***	0.513	***	0.477	***	0.336	***	0.311	***	0.265	***
Th17	RORC	0.103	0.056	0.269	***	0.142	**	0.171	**	0.04	0.456	-0.001	0.980	0.04	0.456
	CCR6	0.369	***	0.181	***	0.355	***	0.274	***	0.098	0.070	0.167	**	0.282	***
	IL17A	0.135	*	0.148	**	0.164	**	0.088	0.104	0.016	0.764	0.08	0.140	0.081	0.134
Treg	FOXP3	0.272	***	0.32	***	0.32	***	0.334	***	0.278	***	0.237	***	0.148	**
	STAT5B	0.62	***	0.66	***	0.585	***	0.631	***	0.52	***	0.561	***	0.367	***
	TGFB1	0.454	***	0.29	***	0.394	***	0.34	***	0.306	***	0.269	***	0.406	***
TAM	CCL2	0.346	***	0.288	***	0.283	***	0.285	***	0.221	***	0.174	**	0.189	***
	IL10	0.313	***	0.267	***	0.344	***	0.306	***	0.284	***	0.261	***	0.273	***
	CD163	0.253	***	0.307	***	0.276	***	0.296	***	0.349	***	0.239	***	0.14	**
	VSIG4	0.2	***	0.2	***	0.239	***	0.272	***	0.281	***	0.199	***	0.175	**
	CSF1R	0.276	***	0.271	***	0.293	***	0.295	***	0.347	***	0.268	***	0.27	***
	FCGR2A	0.34	***	0.293	***	0.403	***	0.374	***	0.39	***	0.325	***	0.377	***
DC	ITGAX	0.43	***	0.325	***	0.391	***	0.385	***	0.311	***	0.354	***	0.398	***
	CD1C	0.324	***	0.234	***	0.295	***	0.265	***	0.154	**	0.191	***	0.155	**
	NRP1	0.605	***	0.566	***	0.566	***	0.512	***	0.52	***	0.395	***	0.342	***
Neutrophils	CCR7	0.296	***	0.258	***	0.34	***	0.279	***	0.148	**	0.165	**	0.098	0.070
	ITGAM	0.267	***	0.263	***	0.313	***	0.334	***	0.403	***	0.395	***	0.379	***
	CD59	0.346	***	0.304	***	0.296	***	0.286	***	0.279	***	0.215	***	0.217	***

CTL: cytotoxic T lymphocytes; TAM: tumor-associated macrophages; DC: dendritic cells.

*P < 0.05, **P < 0.01, ***P < 0.001

## DISCUSSION

Not only cancer genetics, but aberrant epigenetic alternations were reportedly involved in the tumorigenesis and progression of HCC [4]. BRD-containing protein genes, a class of epigenetic readers with unique recognition for N-acetyl-lysine in histones and functions of gene transcription and chromatin modification, have been found to participate in the development of many cancers, including HCC. Although the role of some members of the BRD gene family in HCC has been confirmed, the distinct functions of BRD-containing protein genes in HCC remained to be elucidated. In this study, we performed in-depth analyses on the expressions, mutations and prognostic values of BRD1/2/3/4/7/8/9 in HCC.

Results from our study demonstrated that over-expressions of mRNA and protein were found in BRD1/2/3/4/7/8/9, and mRNA expressions of these genes were significantly correlated with cancer stages and tumor grades in HCC patients. Higher mRNA expressions of BRD3/7/8/9 were significantly related to unfavorable OS of HCC patients, and patients with higher BRD7/8/9 expression levels had significantly shorter DFS. Additionally, multivariate analysis found that high mRNA expressions of BRD4/8/9 were independent prognostic indicators for shorter DFS of HCC patients, and high BRD8 expression also acted as an independent prognostic factor for shorter OS. Moreover, a high rate (52%) of sequence alternations in these seven genes was found in HCC patients, and the genetic alternations in BRD1/2/3/4/7/8/9 were associated with shorter OS and DFS in HCC patients. Further, function and pathway analysis confirmed that BRD-containing proteins genes were mainly involved in regulating the vital processes of gene transcription and chromatin remodeling. Finally, our results also indicated that mRNA expressions of BRD-containing protein genes were significantly associated with diverse immune infiltrating levels and they might play an important role in the HCC immune microenvironment.

BRD1, also known as bromodomain and PHD finger-containing protein 2 (*BRPF2*), acts as a scaffolding subunit in the MOZ-MORF HAT complex [28]. A mechanistic study showed that BRD1 colocalized with sulfatide in HCC cells, promoted integrin αV gene transcription and HCC metastasis by interacting with sulfatide and inducing acetylation. And the knockdown of BRD1 limited the sulfatide-induced acetylation and the occupancy of MOZ on integrin αV gene promoter [29]. In our study, higher mRNA and protein expressions of BRD1 were found in HCC tissues, and mRNA expression of BRD1 was remarkably linked with cancer stages and tumor grades. Although patients with worse OS and DFS tended to express higher BRD1, the statistical difference was not significant. Further researches are needed to assess the oncogenic role of BRD1 in HCC.

BRD2, a member of the bromodomain and extra-terminal (BET) family including mammalian BRD3, BRD4 and BRDT [9], is remarkably over-expressed in breast cancer [30], gastric cancer (GC) [31], malignant pleural mesothelioma (MPM) [32] and castration-resistant prostate cancer (CRPC) [33]. Additionally, higher BRD2 expression was significantly correlated with a detrimental prognosis in breast cancer [30] and CRPC [33]. Studies from Delmore et al. showed that BET proteins could directly up-regulate the expression of certain cancer-related genes, like *c-MYC*, and BET inhibition led to a reduction in cell proliferation by avoiding BET proteins binding to the *MYC* locus [34]. Moreover, in vivo and vitro studies found that the reconstitution of miR-143-3p, which was low-expressed in GC, could inhibit GC cell proliferation and restore its sensitivity to cisplatin by directly down-regulating the expression of the specific downstream target BRD2 and repressing the transcription of *c-MYC* [31]. Our results showed that mRNA and protein expressions of BRD2 were found to be significantly higher in HCC tissues, and mRNA expression of BRD2 was remarkably linked with cancer stages and tumor grades. However, no significant correlation was observed between BRD2 expression and OS or DFS. Further studies are required to evaluate whether BRD2 plays an oncogenic role in HCC.

BRD3, also called *ORFX* or *Fshrg2*, is less well characterized in the BET family. Recently, Welti et al. found that BRD3 mRNA expression was significantly related to androgen receptor-driven transcription in CRPC biopsies [35]. Studies from Zhang et al. revealed that miR-141 could target BRD3 in vitro to reduce apoptosis and promote cell growth, migration and invasion in nasopharyngeal carcinoma cells [36]. Feng et al. demonstrated that BRD3/4 promoted *ESR1* transcription and tamoxifen resistance by interacting with WHSC1 in ER-positive breast cancer [37]. Our results found that higher mRNA and protein expressions of BRD3 were found in HCC, and mRNA expression of BRD3 was remarkably linked with cancer stages and tumor grades.

BRD4 is the most well studied member of the BRD family in multiple cancers, such as melanoma [38], MPM [32], CRPC [35], neuroblastoma [39] and lung adenocarcinoma [40]. Studies carried out by Tsang et al. [41] and Zhang et al. [23] indicated that BRD4 was noticeably up-regulated in HCC tissues and multiple HCC cell lines. Higher BRD4 expression was significantly related to poor prognosis in HCC patients, and BRD4 intensity in cancer tissues was an independent prognostic factor for OS [23]. Mechanistically, over-expression of miR-608 could prevent the proliferation ability of HepG2 cells and HCC growth in mouse xenograft model by down-regulating BRD4 [42]. Moreover, Wang et al. showed that BRD4 promoted HCC cell migration and invasion through inducing matrix metalloproteinase (MMP)-2 and MMP-9, mediated by the Sonic hedgehog signaling pathway [43]. BRD4 inhibition can also suppress the E2F2-cell cycle regulation circuit in HCC cells [44]. Similarly, in this study, higher mRNA and protein expressions of BRD4 were found in HCC, and mRNA expression of BRD4 was significantly correlated with cancer stages and tumor grades. Additionally, BRD4 was also an independent prognostic factor for shorter DFS of HCC patients, suggesting that BRD4 took part in the hepatocarcinogenesis.

BRD7, a subunit of PBAF-specific SWI/SNF complex and functioning as a transcriptional cofactor for tumor suppressor protein p53, has been described as a tumor suppressor gene in many cancers, such as HCC [24], nasopharyngeal carcinoma (NPC) [45] and colorectal cancer (CRC) [46]. Decreased BRD7 expression was correlated with clinical parameters including TNM stage, tumor size, shorter OS and RFS, and was an unfavorable prognostic marker for HCC [24]. In vitro studies showed that HCV facilitated the Ras//Raf/MEK/ERK pathway through a positive feedback regulatory cycle and attenuated the production of BRD7 by a negative feedback mechanism, leading to the facilitation of HCC cell proliferation and HCC development [47]. However, our studies showed the reverse. Higher mRNA and protein expressions of BRD7 were found in HCC, and mRNA expression of BRD7 was correlated with cancer stages and tumor grades. Moreover, increased BRD7 expression was dramatically correlated with shorter OS and DFS in HCC patients. Accordingly, further studies are essential to evaluate the exact role of BRD7 in HCC.

BRD8, an accessory subunit of human NuA4-HAT complex, may function in embryonic or cancer stem cell reprogramming via the *c-MYC* pathway [48]. In mechanism, Yamaguchi et al. suggested that *MRGBP* promoted cancer cell growth in CRC by interacting with BRD8, and inhibiting BRD8 could improve CRC chemotherapy sensitivity [49]. Moreover, Jiang et al. had revealed that miR-185 could attenuate the androgen receptor function indirectly by suppressing BRD8 [50]. In our study, higher mRNA and protein expressions of BRD8 were found in HCC, and mRNA expression of BRD8 was remarkably correlated with cancer stages and tumor grades. Accordingly, higher mRNA expression of BRD8 was also correlated with shorter OS and DFS in HCC patients and was an independent prognostic marker for shorter OS and DFS of HCC, indicating an oncogenic role of BRD8 in HCC.

BRD9, a component of the BAF SWI/SNF complex, has been reported to be over-expressed in MPM [32] and leukemia [51]. Hohmann et al. found that acute myeloid leukemia cells require BRD9 to sustain *MYC* transcription and support proliferation [52]. Over-expression of miR-140-3p negatively affected the tumorigenesis by down-regulating the oncogene BRD9 in squamous cell lung cancer [53]. Conversely, Inoue et al. found that depletion of BRD9 caused melanoma tumorigenesis and it might function as a potent tumor suppressor in melanoma [54]. In our study, higher mRNA and protein expressions of BRD9 were found in HCC, and mRNA expression of BRD9 was significantly correlated with cancer stages and tumor grades. Accordingly, higher mRNA expression of BRD9 was also correlated with shorter OS and DFS in HCC patients and was an independent prognostic marker for poorer DFS of HCC patients, suggesting BRD9 played an oncogenic role in HCC.

Notably, to some extent, this study explored the correlations between BRD-containing protein genes and diverse immune infiltration cells in HCC. The mRNA expression levels of individual BRD genes were significantly positively associated with the immune infiltrating levels of B cells, CD8^+^ T cells, CD4^+^ T cells, macrophages, neutrophils, and DCs. Accordingly, the associations between BRD-containing protein genes and the marker genes of various immune cells (B cells, CTLs, Th1 cells, Th2 cells, Th17 cells, Tregs, TAMs, DCs, and neutrophils) showed a statistical significance, suggesting the critical role of BRD-containing protein genes in the HCC immune microenvironment. In recent years, increasing studies have discovered that BRD-containing protein genes and their inhibitors may function in remodeling the tumor immune microenvironment and suggested that they can be combined with immunotherapies [20–22, 32, 55]. Mishima et al. found that BRD1-mediated HAT activity was crucial for the induction of CD8 expression at several stages of T-lymphocyte development [55]. Treatment of T cells showed that BET inhibitors could maintain CD8^+^ T cells with functional properties of stem cell-like and central memory T cells. And BRD4 could directly up-regulate the expression of the transcription factor BATF in CD8^+^ T cells [20]. To date, there have been limited studies on BRD-containing protein genes in combination with immune-modulatory therapy in HCC. Further researches are required to verify the potential role of BRD-containing protein genes in the HCC immune microenvironment.

The present study had some limitations that should be considered. First, our study of prognostic prediction was mainly retrieved from the HCC patient cohort in TCGA database. Although laboratory experiment procedures could remain consistent from one database, further studies including more independent HCC patient cohorts from other databases are warranted to verify our findings and to explore the clinical use of the BRDs in HCC. Second, results of multivariate survival analysis showed that BRD4/8/9 expressions were independently associated with worse DFS and BRD8 was independently correlated with shorter OS. Therefore, we suggested that BRD4/8/9 were promising candidate biomarkers in HCC, although over-expression of BRD4 had no significant correlation with shorter OS in the GEPIA database and BRD4/9 were not independent predictors of OS in HCC patients. The reason we presumed for these discrepancies might be the existence of confounding factors in our study. In Kaplan-Meier analysis, the influence of an independent variable on a dependent variable contains not only the role of the independent variable itself but also the confounding effect between this variable and other variables. However, in multivariate analysis, a regression model is constructed to adjust potential confounding factors, and thus the real effect of the independent variable could be discovered. In our study, we supposed that the real effect of BRD4 was covered in the GEPIA database and multivariate survival analysis could help to discover its role. However, larger sample sizes and further experiments such as knockdown or over-expression of BRDs on HCC cell or animal models are required. Finally, we did not explore the mechanism of BRD-containing protein genes in HCC and evaluate the potential therapeutic effects between BRDs and anticancer drugs. Recent studies have focused on the selective mechanism of inhibitors toward BRD-containing protein genes such as BRD4 and BRD9 based on molecular dynamics simulations [56, 57]. Further researches are needed to explore the exact mechanism and therapeutic roles of BRDs in HCC.

In conclusion, the current study provided a whole image of the expressions, mutations and prognostic values of different BRD-containing protein genes in HCC. Our study demonstrated that increased expressions of BRD1/2/3/4/7/8/9 were found to be significantly correlated with clinical cancer stages and pathological tumor grades in HCC patients. Moreover, higher mRNA expressions of BRD4/8/9 were promising prognostic indicators in HCC patients. The rate of sequence alternations in BRD1/2/3/4/7/8/9 was relatively high in HCC patients and the genetic alternations were associated with shorter OS and DFS in HCC patients. Thus, BRD4/8/9 could be potential prognostic markers and druggable epigenetic targets in HCC patients.

## MATERIALS AND METHODS

### ONCOMINE database

The mRNA expressions of seven distinct BRD-containing protein genes in various cancer tissues were analyzed using ONCOMINE database (https://www.oncomine.org), which is an integrated publicly accessible online cancer microarray database [58]. Students’ t-test was used to conduct a comparative analysis for transcriptional expression differences of BRD-containing protein genes between normal controls and cancer specimens. The *P*-value was set up at 0.001 and the cut-off of fold change was 1.

### UALCAN analysis

UALCAN (http://ualcan.path.uab.edu), an interactive web-portal based on level 3 RNA-seq and clinical data of 31 cancer types from TCGA datasets, was performed to analyze the relative mRNA expressions of BRD-containing protein genes between HCC and normal samples and their correlation with relative clinicopathologic parameters [59]. The difference of transcriptional expression was compared by students’ t-test and *P*-value < 0.05 was regarded as statistically significant.

### Human Protein Atlas

The Human Protein Atlas (https://www.proteinatlas.org) is a website that contains mRNA and immunohistochemistry (IHC)-based protein expression data from 17 different forms of human cancer [60]. It allows researchers to create an information database of proteins expression patterns in a given tumors of type. In the present study, a direct comparison of protein expressions of different BRD-containing protein genes between normal tissues and HCC tissues was performed by IHC image.

### GEPIA dataset

The prognostic values of individual BRD-containing protein genes in liver cancer were analyzed by using GEPIA (http://gepia.cancer-pku.cn/), which is a web-based tool and capable of providing survival information using 9736 tumors from the TCGA and the GTEx databases, including 364 clinical HCC cases [61]. The cancer patients were categorized into high and low expression groups based on the median values of mRNA expression to analyze overall survival (OS) and disease-free survival (DFS) and validated by Kaplan-Meier survival curves. *P*-value < 0.05 was regarded as statistically significant.

### TCGA database

TCGA provides a huge amount of sequencing and pathological data spanning 33 cancer types [62]. We used the cBioPortal website (http://www.cbioportal.org/) to download clinicopathological data and mRNA sequencing of BRD-containing protein genes in TCGA liver cancer data [63]. After excluding the cases with missing follow-up data, 364 HCC patients were included in the final analysis and their clinicopathological characteristics were summarized in Supplementary Table 2. The effect of clinical characteristics and mRNA expressions of 7 BRD genes on OS and DFS of HCC patients were evaluated using univariate and multivariate Cox proportional hazards model. On multivariate analysis, variables that exhibited an association with *P* ≤ 0.1 in univariate analysis were examined. Statistical analyses were performed using SPSS software version 20.0 (Chicago, IL, USA). Statistical significance was inferred at a *P*-value < 0.05.

### cBioPortal

The cBioPortal (http://www.cbioportal.org/) provides a web resource for exploring, visualizing, and analyzing multidimensional cancer genomics data [63]. From the genomic profiles, we obtained information containing mutations, putative copy-number alternations from GISTIC and mRNA Expression z-Scores (RNA Seq V2 RSEM) with a z-score threshold ± 2.0. Genetic mutations in BRD-containing protein genes and their relationship with OS and DFS of HCC patients were plotted by the Kaplan-Meier method, and significance was analyzed using the log-rank test. *P*-value < 0.05 was regarded as statistically significant.

### STRING database

The STRING database (https://string-db.org/) aims to establish functional protein interaction networks by collecting, scoring and integrating all known protein-protein association information and complementing these with computational predictions [64]. We used it to create an interacting protein network of BRD-containing protein genes that pertained to *Homo sapiens*. Fifty functional interacting partners were displayed with a confidence score > 0.9.

### Gene Ontology (GO) and Kyoto Encyclopedia of Genes and Genomes (KEGG) analysis

Fifty interactors based on the STRING database were analyzed by GO and KEGG in the Database for Annotation, Visualization and Integrated Discovery (DAVID) (https://david.ncifcrf.gov/) [65]. GO enrichment analysis is capable of predicting the functional roles of BRD-containing protein genes and 50 interactors based on 3 GO categories: biological processes (BP), cellular components (CC) and molecular functions (MF). KEGG analysis can predict the pathways associated with the BRD-containing protein genes and 50 functional interactors.

### TIMER database

TIMER (https://cistrome.shinyapps.io/timer/) is a comprehensive website for systematical analysis of immune infiltrates across 32 cancer types [66]. We use TIMER to explore the associations of the expressions of 7 different BRD-containing protein genes with the abundance of immune infiltrates in liver cancer. And correlations between the expressions of 7 distinct BRDs and the gene markers of tumor-infiltrating immune cells were also analyzed. The gene markers used for the analysis of tumor-infiltrating immune cells including B cells, CTLs, Th1 cells, Th2 cells, Th17 cells, Tregs, TAMs, DCs, and neutrophils, were based upon data from previous studies [67, 68].

## Supplementary Material

Supplementary Figure 1

Supplementary Table 1

Supplementary Tables 2, 3, 4 and 5

Supplementary Table 6
