# Supplementary material for: BRD4/8/9 are prognostic biomarkers and associated with immune infiltrates in hepatocellular carcinoma
Source: Aging (Albany NY). 2020 Sep 14;12(17):17541–67. doi: 10.18632/aging.103768 (PMC7521508; doi:10.18632/aging.103768)
Supplement: Supplementary Figure 1 [file aging-12-103768-s004..pdf]

SUPPLEMENTARY FIGURE

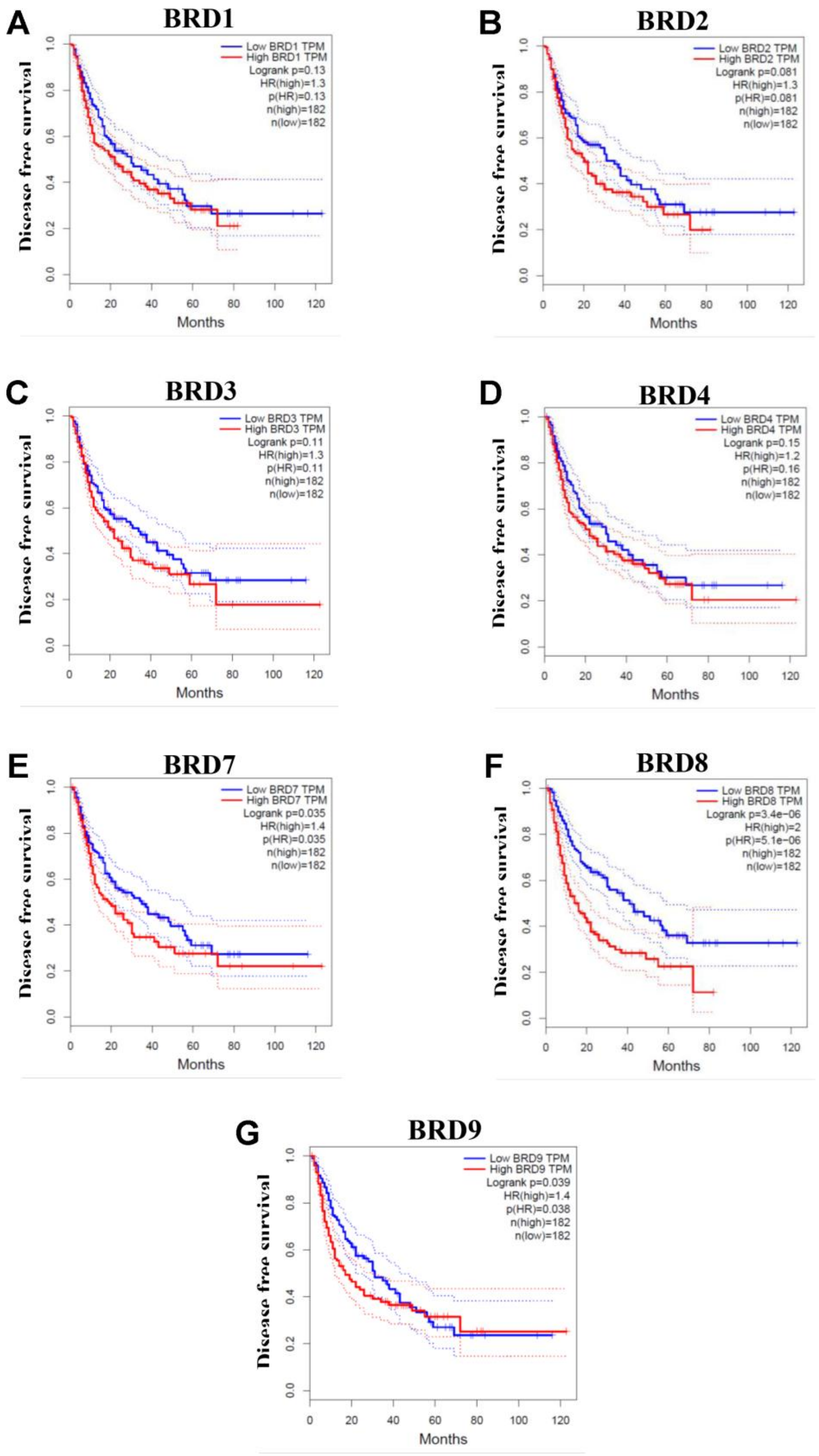

Supplementary Figure 1. Associations between mRNA expression of each BRD-containing protein gene in tumor tissues and DFS of HCC patients (GEPIA). Each mRNA expression of BRD-containing protein gene in tumor tissue was stratified into high or low expression using the median expression value as the cut-off point. The corresponding P-value for Log-rank test in all HCC patients was showed. Higher mRNA expressions of BRD7/8/9 were significantly associated with poorer DFS in HCC patients (**E**–**G**). However, mRNA expressions of BRD1/2/3/4 showed no correlation with prognosis in HCC patients (**A**–**D**).
