# Supplementary material for: BRD4/8/9 are prognostic biomarkers and associated with immune infiltrates in hepatocellular carcinoma
Source: Aging (Albany NY). 2020 Sep 14;12(17):17541–67. doi: 10.18632/aging.103768 (PMC7521508; doi:10.18632/aging.103768)
Supplement: Supplementary Table 1 [file aging-12-103768-s001..docx]

| **Supplementary Table 1. Function of bromodomain-containing proteins.** | | |
| --- | --- | --- |
| **BRD-containing protein gene ID (s)** | **Full name** | **Function** |
| ASH1L | ash1 (absent, small, or homeotic)-like (Drosophila) | Transcriptional activator, methyltransferase |
| ATAD2 | ATPase family, AAA domain containing 2 | ATPase, transcriptional co-activator, implicated in chromatin remodeling and DNA damage response |
| ATAD2B | ATPase family, AAA domain containing 2B | ATPase, co-activator, found at replication sites, consistent with a chromatin-related function |
| BAZ1A | bromodomain adjacent to zinc finger domain 1A | Chromatin assembly and remodeling, implicated in DNA damage response |
| BAZ1B | bromodomain adjacent to zinc finger domain 1B | Chromatin assembly and remodeling, implicated in DNA damage response |
| BAZ2A | bromodomain adjacent to zinc finger domain 2A | Transcriptional repressor, chromatin remodeler |
| BAZ2B | bromodomain adjacent to zinc finger domain 2B | Transcriptional activator, chromatin remodeler |
| BPTF | PHD finger transcription factor | Transcription factor, transcriptional activator, chromatin remodeler and implicated in DNA damage response |
| BRD1 | bromodomain containing 1 | Transcription factor, transcriptional activator, part of the MOZ/MORF acetyltransferase complex |
| BRD2 | bromodomain containing 2 | Transcription factor and transcriptional co-regulator interacting with co-activators, co-repressors, and proteins of the SWI/SNF chromatin remodeling complex, probable additional kinase activity |
| BRD3 | bromodomain containing 2 | Transcription factor and transcriptional co-regulator interacting with co-activators, co-repressors, and proteins of the SWI/SNF chromatin remodeling complex, probable additional kinase activity |
| BRD4 | bromodomain containing 4 | Transcription factor and transcriptional co-regulator, involved in transcriptional plasticity interacting with multiple cell lineage-specific transcription factors and chromatin remodeling factors, histone acetyl transferase through which it promotes chromatin de-compaction, involved in transcriptional response to several cellular stress |
| BRD7 | bromodomain containing 7 | Transcriptional repressor, putative tumour suppressor, within the SWI/SNF chromatin remodeling complex, interacts with p53 and is required for p53-dependent oncogene-induced senescence which prevents tumor growth |
| BRD8 | bromodomain containing 8 | TRAP/TIP60 chromatin remodeling complex component, nuclear receptors co-activator |
| BRD9 | bromodomain containing 9 | Transcriptional activator, subunit of the SWI-SNF chromatin-remodeling complex |
| BRDT | bromodomain, testis-specific | Transcription factor and transcriptional co-regulator interacting with co-activators, co-repressors, and proteins of the SWI/SNF chromatin remodeling complex, probable additional kinase activity |
| BRPF1 | bromodomain and PHD finger containing 1 | Activates components of the MOZ/MORF histone acetyl transferase complexes which function as a transcriptional regulators |
| BRPF3 | bromodomain and PHD finger containing 3 | Activates components of the MOZ/MORF histone acetyl transferase complexes which function as a transcriptional regulators |
| BRWD1 | bromodomain and WD repeat domain containing 1 | Transcriptional activator and chromatin remodeling associated with a component of the SWI/SNF complex |
| BRWD3 | bromodomain and WD repeat domain containing 3 | Chromatin-modifying function involved in JAK/STAT signaling |
| CECR2 | cat eye syndrome chromosome region, candidate 2 | ATP-dependent chromatin remodeling may additionally play a role in DNA damage response |
| CREBBP | CREB binding protein | Transcriptional co-activator of many transcription factors, histone acetyl transferase for histones and non-histones proteins |
| EP300 | E1A binding protein p300 | Transcriptional co-activator of many transcription factors, histone acetyl transferase for histones and non-histones proteins, binds CREB, also co-activator of HIF1A |
| KAT2A | K (lysine) acetyltransferase 2A | Histone acetyl transferase, associates with p300/CBP |
| KAT2B | K (lysine) acetyltransferase 2B | Histone acetyl transferase, associates with p300/CBP |
| KIAA2026 | KIAA2026 | Uncharacterized |
| KTM2A (MLL) | lysine methyltransferase 2A | Transcriptional activator, methyltransferase |
| PBRM1 | polybromo 1 | Subunit of ATP-dependent chromatin-remodeling complexes, component of complexes necessary for ligand-dependent transcriptional activation by nuclear receptors |
| PHIP | pleckstrin homology domain interacting protein | Binds insulin receptor substrate 1 protein and regulates glucose transporter translocation |
| SMARCA2 | SWI/SNF related, matrix associated, actin dependent regulator of chromatin, subfamily a, member 2 | Member of the SWI/SNF family with ATPase and helicase activity to activate gene transcription by modification of chromatin structure |
| SMARCA4 | SWI/SNF related, matrix associated, actin dependent regulator of chromatin, subfamily a, member 4 | Member of the SWI/SNF family with ATPase and helicase activity to activate gene transcription by modification of chromatin structure |
| SP100 | SP100 nuclear antigen | Transcriptional regulators |
| SP110 | SP110 nuclear body protein | Transcriptional regulators |
| SP140 | SP140 nuclear body protein | Transcriptional regulators |
| SP140L | SP140 nuclear body protein-like | Transcriptional regulators |
| TAF1 | TATA-box binding protein associated factor 1 | Participate in basal transcription, serve as co-activators, function in promoter recognition or modify general transcription factors to facilitate complex assembly and transcription initiation. Have two independent protein kinase domains, histone acetyl transferase that can act as a ubiquitin-activating/conjugating enzyme |
| TAF1L | TATA-box binding protein associated factor 1 like | Participate in basal transcription, serve as co-activators, function in promoter recognition or modify general transcription factors to facilitate complex assembly and transcription initiation. Have two independent protein kinase domains, histone acetyl transferase that can act as a ubiquitin-activating/conjugating enzyme |
| TRIM24 | tripartite motif containing 24 | Transcription factor and nuclear receptors co-activator, implicated in DNA damage response |
| TRIM28 | tripartite motif containing 28 | Transcription factor, transcriptional elongation factor, and cofactor |
| TRIM33 | tripartite motif containing 33 | Transcription factor, transcriptional repressor |
| TRIM66 | tripartite motif containing 66 | Transcription factor, transcriptional regulator |
| ZMYND11 | zinc finger, MYND-type containing 11 | Transcriptional repressor implicated in viral infection processes |
| ZMYND8 | zinc finger, MYND-type containing 8 | Transcriptional repressor in complex with histone demethylases; Implicated in DNA damage response |
