# Supplementary material for: BRD4/8/9 are prognostic biomarkers and associated with immune infiltrates in hepatocellular carcinoma
Source: Aging (Albany NY). 2020 Sep 14;12(17):17541–67. doi: 10.18632/aging.103768 (PMC7521508; doi:10.18632/aging.103768)
Supplement: Supplementary Tables 2, 3, 4 and 5 [file aging-12-103768-s003..pdf]

SUPPLEMENTARY TABLES

Supplementary Table 2. Clinicopathological characteristics of 364 HCC patients.

Variables	HCC patients (N = 363)
Age (years), median (IQR)	61 (52-69)
Gender (male/female)	247/117
Weight (kg), median (IQR)	69 (59-82)
PLT (10^9/L), median (IQR)	211 (160.75-299)
Creatinine (mg/dl), median (IQR)	0.9 (0.7-1.1)
Albumin (g/L), median (IQR)	4 (3.5-4.3)
TB (µmol/L), median (IQR)	0.7 (0.5-1.0)
PT (s), median (IQR)	1.1 (1.0-9.25)
AFP (ng/ml), median (IQR)	15 (4-266.25)
Child-Pugh stage	
A	N = 217
B	N = 21
C	N = 1
Adjacent hepatic tissue inflammation	
None	N = 117
Mild	N = 98
Severe	N = 17
Liver fibrosis ishak score category	
No fibrosis	N = 74
Portal fibrosis	N = 31
Fibrous speta	N = 27
Nodular formation and incomplete cirrhosis	N = 9
Established cirrhosis	N = 69
Vascular invasion	
None	N = 204
Micro	N = 90
Macro	N = 16
Histologic grade	
1	N = 54
2	N = 174
3	N = 119
4	N = 12
Pathologic stage	
1	N = 170
2	N = 84
3	N = 83
4	N = 4

IQR: interquartile range; PT: prothrombin time; TB: total bilirubin.

Supplementary Table 3. Univariate analysis for OS and DFS in 364 HCC patients.

Variables	OS		DFS	
	HR (95% CI)	*P*-value	HR (95% CI)	*P*-value
Age (years)	1.012 (0.999-1.026)	0.076	0.998 (0.986-1.010)	0.735
Gender	1.236 (0.866-1.766)	0.243	1.126 (0.817-1.553)	0.469
Weight (kg)	0.993 (0.983-1.003)	0.175	0.999 (0.991-1.006)	0.705
PLT (10^9/L)	1.000 (1.000-1.000)	0.735	1.000 (1.000-1.000)	0.759
Creatinine (mg/dl)	1.002 (0.986-1.018)	0.794	1.002 (0.986-1.017)	0.830
Albumin (g/L)	0.987 (0.945-1.032)	0.576	0.999 (0.995-1.003)	0.668
TB (μmol/L)	0.975 (0.845-1.124)	0.723	1.047 (0.960-1.141)	0.299
PT (s)	1.015 (0.978-1.055)	0.432	1.002 (0.970-1.034)	0.919
AFP (ng/ml)	1.000 (1.000-1.000)	0.432	1.000 (1.000-1.000)	0.282
Child-Pugh stage	1.523 (0.836-2.775)	0.170	1.250 (0.729-2.143)	0.418
Adjacent hepatic tissue inflammation	1.158 (0.797-1.683)	0.441	1.170 (0.869-1.575)	0.302
Liver fibrosis ishak score category	0.930 (0.800-1.080)	0.342	1.049 (0.937-1.174)	0.408
Vascular invasion	1.384 (1.001-1.914)	0.050	1.687 (1.288-2.210)	0.000
Histologic grade	1.123 (0.888-1.422)	0.333	1.103 (0.904-1.347)	0.334
Pathologic stage	1.660 (1.355-2.035)	0.000	1.731 (1.450-2.066)	0.000
BRD1	1.287 (0.909-1.823)	0.155	1.224 (0.905-1.654)	0.189
BRD2	1.282 (0.905-1.816)	0.162	1.479 (1.092-2.003)	0.011
BRD3	1.127 (0.797-1.593)	0.498	1.213 (0.898-1.639)	0.209
BRD4	1.385 (0.977-1.962)	0.067	1.608 (1.187-2.177)	0.002
BRD7	1.472 (1.040-2.083)	0.029	1.227 (0.908-1.658)	0.182
BRD8	1.913 (1.344-2.723)	0.000	1.905 (1.404-2.585)	0.000
BRD9	1.686 (1.188-2.394)	0.003	1.794 (1.323-2.434)	0.000

HR: hazard ratio

Supplementary Table 4. Multivariate analysis for OS in 364 HCC patients.

Variables	Multivariate[a]		Multivariate[b]		Multivariate[c]		Multivariate[d]	
	HR (95% CI)	*P*-value	HR (95% CI)	*P*-value	HR (95% CI)	*P*-value	HR (95% CI)	*P*-value
Age	1.016 (0.999-1.033)	0.065	1.015 (0.998-1.032)	0.079	1.015 (0.998-1.032)	0.081	1.015 (0.998-1.032)	0.079
Vascular invasion	1.077 (0.750-1.549)	0.687	1.107 (0.772-1.586)	0.582	1.078 (0.755-1.540)	0.679	1.097 (0.766-1.572)	0.613
Pathologic stage	1.483 (1.149-1.914)	0.002	1.476 (1.145-1.902)	0.003	1.496 (1.160-1.930)	0.002	1.467 (1.133-1.898)	0.004
BRD4	1.337 (0.867-2.063)	0.189						
BRD7			1.357 (0.884-2.085)	0.163				
BRD8					1.545 (1.007-2.369)	0.046		
BRD9							1.244 (0.800-1.933)	0.332

[a]Multivariate analysis for age, vascular invasion, pathologic stage and BRD4.
[b]Multivariate analysis for age, vascular invasion, pathologic stage and BRD7.
[c]Multivariate analysis for age, vascular invasion, pathologic stage and BRD8.
[d]Multivariate analysis for age, vascular invasion, pathologic stage and BRD9.

Supplementary Table 5. Multivariate analysis for DFS in 364 HCC patients.

Variables	Multivariate[a]		Multivariate[b]		Multivariate[c]		Multivariate[d]	
	HR (95% CI)	*P*-value	HR (95% CI)	*P*-value	HR (95% CI)	*P*-value	HR (95% CI)	*P*-value
Vascular invasion	1.221 (0.897-1.664)	0.205	1.231 (0.905-1.673)	0.185	1.237 (0.911-1.681)	0.173	1.274 (0.937-1.733)	0.122
Pathologic stage	1.590 (1.270-1.989)	0.000	1.546 (1.234-1.936)	0.000	1.568 (1.252-1.964)	0.000	1.506 (1.197-1.894)	0.000
BRD2	1.411 (0.982-2.027)	0.063						
BRD4			1.609 (1.119-2.313)	0.010				
BRD8					1.703 (1.191-2.435)	0.004		
BRD9							1.590 (1.103-2.291)	0.013

[a]Multivariate analysis for vascular invasion, pathologic stage and BRD2.
[b]Multivariate analysis for vascular invasion, pathologic stage and BRD4.
[c]Multivariate analysis for vascular invasion, pathologic stage and BRD8.
[d]Multivariate analysis for vascular invasion, pathologic stage and BRD9.
