# Supplementary material for: BRD4/8/9 are prognostic biomarkers and associated with immune infiltrates in hepatocellular carcinoma
Source: Aging (Albany NY). 2020 Sep 14;12(17):17541–67. doi: 10.18632/aging.103768 (PMC7521508; doi:10.18632/aging.103768)
Supplement: Supplementary Table 6 [file aging-12-103768-s002..docx]

| **Supplementary Table 6. Summary of significantly enriched GO annotations of BRD-containing protein genes related network.** | | | |
| --- | --- | --- | --- |
| **Description** | **Gene Count** | **P-value** | **Enriched genes** |
| ***Biological Process*** |  |  |  |
| chromatin remodeling | 22 | 6.55E-35 | MORF4L1, KAT2B, MORF4L2, ARID1A, ARID1B, DMAP1, HDAC2, HDAC1, SMARCE1, SMARCD2, SMARCD3, SMARCB1, SMARCC1, SMARCD1, SMARCC2, PBRM1, RUVBL2, ACTL6A, BRD4, RUVBL1, SMARCA2, SMARCA4 |
| transcription, DNA-templated | 44 | 1.79E-29 | ING5, MORF4L1, DPF3, MEAF6, ING3, MORF4L2, DMAP1, TRRAP, ARID2, VPS72, JADE1, EPC1, BRPF1, SMARCD2, SMARCB1, SMARCD3, BRD7, PBRM1, ACTL6A, BRD4, BRD9, BRD8, YEATS4, KAT7, BRD2, BRD3, TP53, ARID1A, PHF12, ARID1B, MRGBP, KAT5, SS18, HDAC2, HDAC1, SMARCC1, SMARCC2, RUVBL2, RUVBL1, KAT6B, SMARCA2, APBB1, KAT6A, SMARCA4 |
| histone H2A acetylation | 13 | 2.41E-28 | MORF4L1, YEATS4, MEAF6, ING3, MORF4L2, TRRAP, DMAP1, EPC1, ACTL6A, RUVBL2, RUVBL1, EP400, BRD8 |
| covalent chromatin modification | 19 | 3.31E-26 | DPF3, BRD2, ING3, BRD3, ARID1A, ARID1B, ARID2, VPS72, SMARCE1, SMARCD2, SMARCD3, SMARCB1, SMARCC1, SMARCD1, SMARCC2, PBRM1, BRD4, BRD9, SMARCA4 |
| histone H4 acetylation | 14 | 2.76E-25 | MORF4L1, YEATS4, ING3, MORF4L2, TRRAP, DMAP1, EPC1, EP300, ACTL6A, RUVBL2, RUVBL1, APBB1, EP400, BRD8 |
| positive regulation of transcription, DNA-templated | 18 | 4.69E-13 | ING5, YEATS4, TP53, ARID1A, KAT5, EPC1, BRPF1, HDAC2, HDAC1, SMARCD3, SMARCC1, SMARCC2, BRD7, KAT6B, APBB1, SMARCA2, KAT6A, SMARCA4 |
| regulation of transcription from RNA polymerase II promoter | 17 | 6.22E-13 | BRD2, BRD3, ARID1A, MRGBP, SMARCE1, SMARCD2, SMARCD3, SMARCB1, SMARCC1, SMARCD1, SMARCC2, BRD7, ACTL6A, RUVBL1, SMARCA2, SMARCA4, BRD8 |
| positive regulation of transcription from RNA polymerase II promoter | 20 | 1.67E-10 | KAT2B, MORF4L2, TP53, KAT5, JADE1, SS18, EPC1, EP300, HDAC2, HDAC1, SMARCB1, SMARCC1, H2AFZ, RUVBL2, BRD4, KAT6B, SMARCA2, APBB1, BRD8, SMARCA4 |
| negative regulation of transcription, DNA-templated | 15 | 6.46E-10 | ING4, TP53, DMAP1, PHF12, KAT5, EPC1, HDAC2, SMARCE1, HDAC1, SMARCC2, BRD7, KAT6B, SMARCA2, KAT6A, SMARCA4 |
| regulation of transcription, DNA-templated | 23 | 8.24E-10 | MORF4L1, YEATS4, MEAF6, DPF3, KAT7, ING3, MORF4L2, TP53, TRRAP, KAT5, ARID2, EP300, HDAC1, PBRM1, RUVBL2, RUVBL1, KAT6B, APBB1, SMARCA2, BRD9, KAT6A, BRD8, SMARCA4 |
| ***Cellular Component*** |  |  |  |
| NuA4 histone acetyltransferase complex | 15 | 1.94E-33 | MORF4L1, YEATS4, MEAF6, ING3, MORF4L2, TRRAP, DMAP1, KAT5, MRGBP, EPC1, ACTL6A, RUVBL2, RUVBL1, EP400, BRD8 |
| nucleoplasm | 47 | 5.99E-30 | ING5, MORF4L1, ING4, MEAF6, ING3, MORF4L2, DMAP1, TRRAP, ARID2, VPS72, JADE1, EPC1, JADE2, BRPF1, SMARCD2, SMARCB1, SMARCD3, BRD7, PBRM1, ACTL6A, BRD4, BRD8, BRD1, YEATS4, KAT7, KAT2B, TP53, ARID1A, PHF12, ARID1B, MRGBP, KAT5, ATM, HDAC2, EP300, HDAC1, SMARCE1, SMARCC1, SMARCC2, RUVBL2, RUVBL1, KAT6B, SMARCA2, APBB1, EP400, KAT6A, SMARCA4 |
| SWI/SNF complex | 12 | 7.04E-26 | SMARCD2, SMARCE1, SMARCB1, SMARCD3, SMARCC1, SMARCC2, SMARCD1, ARID1A, ACTL6A, ARID1B, SMARCA2, SMARCA4 |
| npBAF complex | 11 | 1.42E-24 | SS18, SMARCE1, SMARCB1, SMARCD3, SMARCC1, SMARCC2, SMARCD1, ARID1A, ACTL6A, SMARCA2, SMARCA4 |
| nBAF complex | 11 | 2.14E-23 | DPF3, SMARCE1, SMARCB1, SMARCD3, SMARCC1, SMARCC2, SMARCD1, ARID1A, ARID1B, SMARCA2, SMARCA4 |
| histone acetyltransferase complex | 8 | 3.17E-13 | JADE1, MEAF6, ING4, JADE2, KAT7, JADE3, EP300, TRRAP |
| nuclear chromatin | 13 | 3.28E-13 | HDAC2, SMARCD2, SMARCE1, HDAC1, SMARCB1, SMARCD3, SMARCC1, SMARCC2, TP53, ARID1A, ACTL6A, SMARCA2, SMARCA4 |
| nucleus | 41 | 2.09E-11 | ING5, MORF4L1, ING4, ING3, MORF4L2, TRRAP, DMAP1, VPS72, JADE1, EPC1, BRPF1, SMARCD3, SMARCB1, H2AFZ, BRD7, ACTL6A, BRD4, BRD8, BRD1, KAT7, BRD2, KAT2B, BRD3, TP53, ARID1A, PHF12, MRGBP, KAT5, ATM, SS18, HDAC2, EP300, HDAC1, SMARCE1, RUVBL2, RUVBL1, KAT6B, SMARCA2, APBB1, KAT6A, SMARCA4 |
| protein complex | 12 | 2.76E-8 | HDAC2, SMARCD2, SMARCE1, HDAC1, SMARCB1, SMARCC1, SMARCC2, TP53, ACTL6A, APBB1, VPS72, SMARCA4 |
| nucleolus | 9 | 0.003530268 | MEAF6, ING3, KAT7, SMARCB1, MORF4L2, TP53, KAT5, SMARCA4, KAT6A |
| ***Molecular Function*** |  |  |  |
| chromatin binding | 21 | 2.82E-19 | ING5, MORF4L1, BRD2, KAT2B, BRD3, TP53, PHF12, HDAC2, EP300, SMARCE1, HDAC1, SMARCD3, SMARCC1, SMARCD1, SMARCC2, PBRM1, ACTL6A, BRD4, APBB1, SMARCA2, EP400 |
| transcription coactivator activity | 18 | 1.61E-18 | ING4, KAT2B, ARID1A, ARID1B, KAT5, EP300, SMARCE1, SMARCD2, SMARCD3, SMARCB1, SMARCC1, SMARCD1, SMARCC2, BRD7, ACTL6A, SMARCA2, KAT6A, SMARCA4 |
| RNA polymerase II distal enhancer sequence-specific DNA binding | 11 | 8.86E-15 | HDAC2, SMARCD2, SMARCE1, HDAC1, SMARCB1, SMARCC1, SMARCC2, H2AFZ, RUVBL2, ACTL6A, SMARCA4 |
| nucleosomal DNA binding | 10 | 2.09E-14 | HDAC2, SMARCD2, SMARCE1, HDAC1, SMARCB1, SMARCC1, SMARCC2, H2AFZ, ACTL6A, SMARCA4 |
| histone acetyltransferase activity | 8 | 1.74E-10 | EPC1, ING3, KAT7, KAT2B, EP300, KAT6B, KAT5, KAT6A |
| protein binding | 51 | 6.13E-10 | MORF4L1, MORF4L2, DMAP1, EPC1, JADE1, JADE2, BRPF1, JADE3, BRPF3, SMARCD2, SMARCD1, BRD7, H2AFZ, BRD4, BRD9, TP53, ARID1A, ARID1B, SS18, EP300, SMARCE1, RUVBL2, RUVBL1, SMARCA2, EP400, SMARCA4, ING5, MEAF6, ING4, TRRAP, ARID2, VPS72, SMARCB1, PBRM1, ACTL6A, YEATS4, BRD2, KAT7, KAT2B, BRD3, PHF12, MRGBP, KAT5, ATM, HDAC2, HDAC1, SMARCC1, SMARCC2, KAT6B, APBB1, KAT6A |
| transcription factor binding | 11 | 2.33E-8 | KAT2B, HDAC2, EP300, HDAC1, SMARCD3, TP53, BRD7, KAT6B, APBB1, SMARCA4, KAT6A |
| RNA polymerase II core promoter proximal region sequence-specific DNA binding | 10 | 1.97E-6 | HDAC2, SMARCD2, SMARCE1, HDAC1, SMARCB1, SMARCC1, SMARCC2, H2AFZ, ACTL6A, SMARCA4 |
| zinc ion binding | 16 | 3.16E-6 | ING5, ING4, DPF3, BRD1, ING3, KAT7, TP53, PHF12, JADE1, BRPF1, JADE2, EP300, JADE3, BRPF3, KAT6B, KAT6A |
| DNA binding | 17 | 5.68E-5 | YEATS4, TP53, ARID1A, ARID1B, ARID2, VPS72, ATM, BRPF1, EP300, SMARCE1, SMARCB1, SMARCC1, SMARCC2, PBRM1, KAT6B, EP400, KAT6A |
| ***KEGG Pathway*** |  |  |  |
| Thyroid hormone signaling pathway | 5 | 3.31E-5 | KAT2B, HDAC2, EP300, HDAC1, TP53 |
| HTLV-I infection | 6 | 4.22E-5 | KAT2B, EP300, TP53, TRRAP, KAT5, ATM |
| Cell cycle | 5 | 4.45E-5 | HDAC2, EP300, HDAC1, TP53, ATM |
| Notch signaling pathway | 4 | 6.71E-5 | KAT2B, HDAC2, EP300, HDAC1 |
| Transcriptional misregulation in cancer | 5 | 1.42E-4 | SS18, HDAC2, HDAC1, TP53, ATM |
| Viral carcinogenesis | 5 | 3.14E-4 | KAT2B, HDAC2, EP300, HDAC1, TP53 |
| Huntington’s disease | 4 | 0.003909615 | HDAC2, EP300, HDAC1, TP53 |
| Chronic myeloid leukemia | 3 | 0.006663097 | HDAC2, HDAC1, TP53 |
| Epstein-Barr virus infection | 3 | 0.018331589 | HDAC2, HDAC1, TP53 |
| Wnt signaling pathway | 3 | 0.023116873 | EP300, TP53, RUVBL1 |
| Pathways in cancer | 4 | 0.027669822 | HDAC2, EP300, HDAC1, TP53 |
| Alcoholism | 3 | 0.036680203 | HDAC2, HDAC1, H2AFZ |
